# Monitoring of treatment with low molecular weight heparins using viscoelastic devices

**DOI:** 10.1186/cc13285

**Published:** 2014-03-17

**Authors:** U Schött, A Larsson, O Thomas, N Tynngård

**Affiliations:** 1 Lund University, Lund, Sweden; 2 Linköping University, Linköping, Sweden

## Introduction

Few studies have investigated the use of viscoelastic devices for monitoring of treatment with LMWHs and to our knowledge there are no studies comparing different LMWHs or different visocoelastic methods.

## Methods

Enoxaparin (Klexane) and tinzaparin (Innohep) were added to 2 ml citrated blood from 10 intensive care patients to obtain plasma concentrations of 0, 0.5, 1.0 and 1.5 IU/ml enoxaparin and tinzaparin, respectively. The study was approved by the local ethics committee and with written consent (relatives). Clot formation and clot retraction was studied using ROTEM and ReoRox.

## Results

ROTEM analysis showed prolonged clot formation (CT) with increasing concentrations of enoxaparin and tinzaparin (more so). ReoRox analysis showed that the initiation of clot formation (COT1) increased with increasing doses of enoxaparin and tinzaparin (more so), as did the progression of clot formation (COT2 - COT1), thus resulting in a prolongation until complete clot formation (COT2). See Table 1.

**Table 1:** ROTEM and FOR variables for enoxaparin and tinzaparin

		Enoxaparin			Tinzaparin		
			
	0 IU/ml	0.5 IU/ml	1.0 IU/ml	1.5 IU/ml	0.5 IU/ml	1.0 IU/ml	1.5 IU/ml
ROTEM							
CT (seconds)	175 ± 38	191 ± 89*	214 ± 109	249 ± 94*	223 ± 53*	289 ± 84*	326 ± 125*
CFT (seconds)	77 ± 23	87 ± 21	85 ± 16	83 ± 22	74 ± 27	84 ± 45	80 ± 21
Angle (°)	75 ± 4	74 ± 4	75 ± 3	74 ± 4	75 ± 5	73 ± 5	73 ± 4
MCF (mm)	62 ± 5	61 ± 7	63 ± 5	63 ± 6	68 ± 7*	64 ± 8	65 ± 6*
MCL (%)	8 ± 4	6 ± 3+	5 ± 4	2 ± 5*	5 ± 4*	6 ± 4	2 ± 4*
ReoRox							
COT1 (seconds)	30 ± 5	35 ± 7*	39 ± 8*	38 ± 11*	36 ± 4*	45 ± 9*	48 ± 15*
COT2 (seconds)	65 ± 11	76 ± 13*	82 ± 14*	85 ± 20*	74 ± 5*	91 ± 16*	108 ± 29*
COT2 - COT1 (seconds)	34 ± 10	41 ± 8*	43 ± 9*	46 ± 10*	37 ± 4	46 ± 11*	51 ± 15*

Data presented as median (SD). *P < 0.05 compared with 0 IU/ml LMWH.

## Conclusion

Clot initiation was prolonged with both drugs and detected by both ROTEM and ReoRox. Clot formation was more decreased with tinzaparin than enozaparin and only detected by ReoRox.

